# Emotion Recognition Using Multi-View EEG-fNIRS and Cross-Attention Feature Fusion

**DOI:** 10.3390/bios16030145

**Published:** 2026-03-02

**Authors:** Ni Yan, Guijun Chen, Xueying Zhang

**Affiliations:** College of Electronic Information Engineering, Taiyuan University of Technology, Taiyuan 030024, China; 2023521562@link.tyut.edu.cn (N.Y.); zhangxy@tyut.edu.cn (X.Z.)

**Keywords:** emotion recognition, multi-view, electroencephalogram (EEG), functional near-infrared spectroscopy (fNIRS), cross-attention

## Abstract

To improve the accuracy of emotion recognition, this paper proposes a multi-view EEG-fNIRS and cross-attention fusion module named FGCN-TCNN-CAF, which employs a differentiated modeling strategy for the frequency, spatial, and temporal features of EEG-fNIRS signals. First, frequency-domain and time-domain features are extracted from EEG, and time-domain features are obtained from fNIRS signals. Then, a frequency-domain graph convolutional network (FGCN) and a time-domain convolutional network (TCNN) are deployed in parallel. The EEG feature views from different frequency bands are modeled using an FGCN module to capture graph-structured relationships, while the time-domain views of EEG and fNIRS are processed by a TCNN module to extract spatial and temporal features. Finally, a cross-attention fusion network (CAF) is applied to achieve interactive fusion of multimodal features. Experiments demonstrate that the proposed multi-view EEG approach achieves higher recognition accuracy compared to using only the EEG view. Additionally, the mmultimodalrecognition results outperform single-modal EEG and single-modal fNIRS by 1.73% and 6.65%, respectively. When compared with other emotion recognition models, the proposed method achieves the highest accuracy of 96.09%, proving its superior performance.

## 1. Introduction

### 1.1. Background

Emotional expression is a crucial component of human life and learning, playing a significant role in cognition, decision-making, and interpersonal communication [1]. With the advancement of artificial intelligence technology, emotion recognition has demonstrated broad application prospects in fields such as human–computer interaction and medical assistance [2]. Traditional emotion recognition research relies on non-physiological signals such as facial expressions [3], body movements [4], and speech signals [5], which are susceptible to subjective influences. In contrast, physiological signals can objectively and authentically reflect emotional states.

Common physiological signals include electroencephalography (EEG) and functional near-infrared spectroscopy (fNIRS) [6]. EEG offers high temporal resolution and can dynamically capture transient changes in neural electrical activity, whereas fNIRS provides relatively high spatial resolution and reflects localized metabolic changes in the cerebral cortex [7]. Both modalities are related to brain activity, and their joint analysis can help reveal the dynamic evolution of emotions, which is of significant research value for affective recognition tasks [8]. However, EEG and fNIRS differ markedly in channel spatial layouts and signal generation mechanisms, which poses challenges for multimodal fusion, such as modeling non-Euclidean channel relationships and insufficient cross-modal interaction, and has become a key issue in current research.

### 1.2. Related Work

Early emotion recognition primarily relied on methods such as support vector machine (SVM) [9], random forest (RF) [10], and k-nearest neighbor (KNN) [11] to construct models. With the advancement of deep learning, convolutional neural networks (CNN) [12] and graph convolutional neural networks (GCN) [13] have been successively applied to EEG-based emotion recognition research. For EEG data, one line of research employs CNN-based methods to automatically learn spatiotemporal features from multi-channel signals. For example, Zhai et al. [14] proposed a novel lightweight adaptive dynamic focusing on the convolutional neural network (LAND-FCNN), which integrates partial convolution sampling and multi-class interactive attention mechanisms. Zhong et al. [15] utilized tunable Q-factor wavelet transform for feature extraction to obtain effective patterns of EEG signals and constructed a novel spatiotemporal representation for EEG signals based on CNN and long short-term memory (LSTM), achieving a recognition rate of 95.33% on the SEED dataset. Zhao et al. [16] proposed CTNet, which uses a CNN to extract local features and a Transformer encoder to learn global representations, achieving high recognition accuracy. Wang et al. [17] proposed a two-dimensional convolutional neural network that employs two convolution kernels of different sizes to extract emotion-related features along the temporal and spatial dimensions. On the DEAP dataset, the best accuracies for binary classification of arousal and valence can reach 99.99% and 99.98%, respectively. Wang et al. [18] proposed ST-CapsNet, which enhances event-related information by incorporating spatial and temporal attention and combines it with a capsule network to learn discriminative features for emotion recognition.

On the other hand, since the channel arrangement of EEG and fNIRS signals belongs to non-Euclidean space, CNN cannot effectively model the inter-channel correlations when processing such data. In contrast, GCN can directly handle non-Euclidean spatial data by introducing graph nodes and edges to model complex connection patterns among different signal channels [19]. Song et al. [20] proposed a dynamic graph convolutional neural network (DGCNN), which dynamically learns connectivity relationships between electrodes and extracts more discriminative EEG features, achieving a recognition accuracy of 90.4% on the SEED dataset. Du et al. [21] proposed a multi-dimensional graph convolutional network (MD-GCN) using graph-structured representations that incorporate channel and spatial information, enabling effective emotion recognition with an accuracy of 92.15% on the SEED dataset. Jin et al. [22] introduced a pyramid graph convolutional network (PGCN), which aggregates features at three different hierarchical levels using GCN and divides the brain into two distinct regions based on prior knowledge of brain structure, focusing on functional connectivity within these regions. This approach achieved a recognition performance of 96.93% on the SEED dataset.

fNIRS characterizes metabolic activity in the cerebral cortex by measuring changes in blood oxygenation and has been increasingly used for decoding emotional states in recent years. Si et al. [23] developed a dual-branch joint network (DBJNet) to decode fNIRS-based emotional states and effectively distinguish among three emotion categories. Ding et al. [24] proposed a multi-scale feature fusion approach that integrates vector-based features and time-delay embedding features for emotion recognition. Si et al. [25] further proposed the HSCTN model, which uses convolutional modules to extract spatiotemporally fused features and employs a spatial transformer module to learn channel-wise attention.

By combining EEG and fNIRS, their complementary temporal and spatial resolutions can be leveraged to capture emotion-related features more comprehensively [26]. Common fusion strategies mainly fall into feature-level fusion and model-level fusion. Feature-level fusion typically concatenates low-level features directly. For example, Chen et al. [27] aligned EEG and fNIRS signals along the temporal dimension and fused them along the channel dimension, then fed the fused representation into a TC-ResNet model composed of a temporal convolutional network and a residual network to model temporal features. Their results showed that, compared with unimodal inputs, bimodal fusion can achieve higher overall classification accuracy. Chen et al. [28] proposed GCN-CA-CapsNet, which fuses EEG–fNIRS features via graph convolution on a Pearson-correlation-based adjacency matrix and combines graph convolution with a capsule attention network for EEG–fNIRS analysis, achieving an accuracy of 97.91%. In contrast, model-level fusion allows each modality to be processed through a deeper, modality-specific pathway and then interacts in a higher-level semantic space. For instance, Pandey et al. [29] proposed fNIRSNET to process fNIRS signals, which employs CNN to generate and fuse multi-view spatiotemporal representations. The network was further extended by integrating it with an EEG network to achieve multimodal classification.

Overall, existing unimodal EEG- based or fNIRS-based methods have achieved relatively high accuracy on some datasets. However, EEG–fNIRS fusion for affective recognition still faces three main limitations. First, the non-Euclidean channel topology is not sufficiently modeled. The spatial layout of EEG channels exhibits non-Euclidean characteristics, and the functional connectivity among channels is complex with long-range dependencies; relying solely on Euclidean architectures such as CNNs makes it difficult to adequately capture such topological relationships [10]. Second, the interaction mechanism is often weak. EEG reflects rapid neural electrical activity, whereas fNIRS reflects slower hemodynamic and metabolic responses. The substantial differences in temporal dynamics and signal generation mechanisms limit effective cross-modal collaborative modeling. Third, structured multi-view joint modeling is still lacking. Many multimodal approaches struggle to jointly couple frequency-domain information, inter-channel relationships, and spatiotemporal features, which prevents complementary information from being fully exploited. Therefore, it is of significant research importance to develop a model that can characterize non-Euclidean channel relationships, extract multi-view features in both the time and frequency domains, and achieve efficient fusion through stronger cross-modal interaction mechanisms.

### 1.3. The Contributions of This Study

To address these limitations, this study proposes a multi-view EEG-fNIRS and cross-attention fusion network model named FGCN-TCNN-CAF using frequency-domain graph convolutional network, time domain convolutional neural network and cross-attention fusion network, which is based on the neurophysiological mechanisms of EEG-fNIRS signals and employs differentiated modeling approaches. The main contributions of this paper are as follows.

1. EEG frequency-domain topology modeling: A frequency-domain graph convolutional network (FGCN) is proposed. The network constructs a learnable adjacency matrix based on prior knowledge and ensures training stability for deep networks through residual connections. Furthermore, it employs a graph attention mechanism to learn inter-channel weights adaptively, obtaining a channel-interaction graph-structured representation across five frequency bands and providing a more discriminative frequency-domain representation for subsequent fusion.
2. Spatiotemporal convolutional architecture: A spatial–temporal convolutional neural network (TCNN) is introduced. The spatial convolution component incorporates two local convolutional kernels and one global convolutional kernel, aiming to enhance the model’s spatial perception capability. Subsequently, multi-scale temporal convolutions are employed to model temporal dynamic variations, enabling spatiotemporal feature extraction from time-series signals.
3. Cross-attention fusion: A cross-attention fusion network (CAF) is utilized to achieve triple interactive fusion between frequency-domain channel-interaction features and time-domain spatiotemporal dynamic responses. This approach fully exploits the complementary information between EEG and fNIRS signals.

The remainder of this paper is organized as follows. Section 2 presents the materials and methods. Section 3 provides the experimental results and analysis. Section 4 discusses the paper and Section 5 concludes the paper.

## 2. Materials and Methods

### 2.1. EEG-fNIRS Dataset

**Figure 1:**
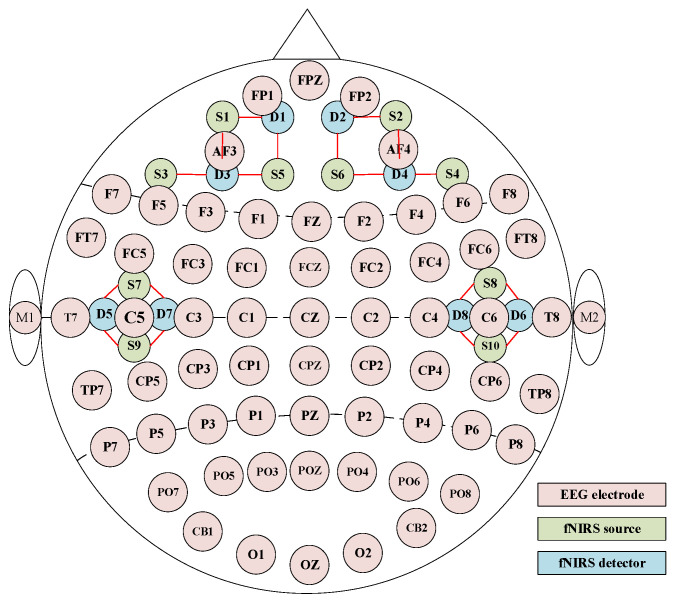
EEG and fNIRS channel distribution map.

The EEG-fNIRS emotion dataset utilized in this study is the ENTER dataset [28]. The emotional stimuli comprised 60 carefully selected video clips, each approximately 1–2 min in duration, covering four emotional categories: happiness, sadness, fear, and neutral. Each emotion category includes 15 video clips. Fifty university students (25 males and 25 females) voluntarily participated. Each participant watched all 60 video clips and completed a 30 s emotion assessment after each clip. Data acquisition followed the international 10–20 system, with synchronous recording of 64-channel EEG data at 1000 Hz and 18-channel fNIRS data at 11 Hz. The detailed channel distribution map is illustrated in Figure 1. Further dataset details are available at https://gitee.com/tycgj/enter, accessed on 12 June 2025.

Since EEG and fNIRS signals are susceptible to environmental noise and other physiological artifacts, preprocessing is generally required prior to feature extraction. For EEG signals, preprocessing was performed using the EEGLAB v2023.0 toolbox. The signals were first re-referenced to the bilateral mastoids (M1, M2). Baseline correction was then applied based on the 2 s interval preceding each trial to eliminate baseline drift. Subsequently, bandpass filtering was conducted to retain the effective frequency band of 0.5–50 Hz. Finally, independent component analysis (ICA) was employed to remove artifact components such as electrooculography (EOG) and electromyography (EMG).

For fNIRS signals, the preprocessing procedure included baseline correction using the 2 s period prior to stimulus onset, followed by bandpass filtering within the 0.01–0.2 Hz frequency range. Segments exhibiting significant abrupt changes were identified and removed. Subsequently, the NirSpark v2.1 software was utilized to convert the raw optical density signals into relative concentration changes in oxygenated hemoglobin (HbO) and deoxygenated hemoglobin (HbR) based on the modified Beer–Lambert law [30].

### 2.2. FGCN-TCNN-CAF Module

**Figure 2:**
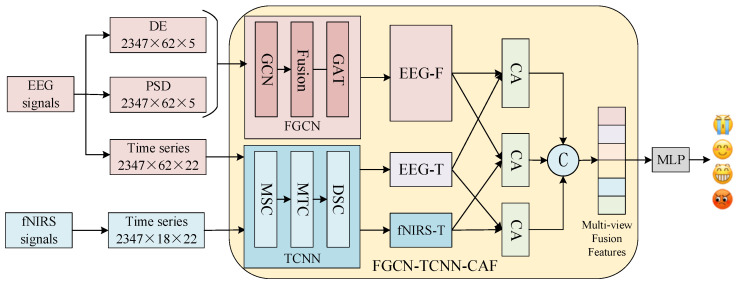
Schematic of the proposed FGCN-TCNN-CAF framework. Abbreviations: GCN, graph convolutional network; GAT, graph attention network; MSC, multi-scale spatial convolution; MTC, multi-scale temporal convolution; DSC, depthwise separable convolution; EEG-F, EEG frequency-domain features; EEG-T, EEG spatial–temporal features; fNIRS-T, fNIRS spatial–temporal features; CA, cross-attention network; MLP, multilayer perceptron.

In the FGCN-TCNN-CAF module, frequency-domain features and time-domain features of EEG signals are first extracted separately, along with time-domain features of fNIRS signals. Subsequently, the FGCN and TCNN module are introduced in parallel. EEG feature views from different frequency bands are modeled for graph-structured relationships via FGCN module, while time-domain views of EEG and fNIRS signals undergo spatiotemporal feature extraction through TCNN module. Finally, the CAF module performs deep interactive fusion of the extracted features. The overall architecture of the module is illustrated in Figure 2. Our proposed module comprehensively integrates multi-level information, including spatiotemporal, frequency, and inter-channel correlations of EEG-fNIRS signals, thereby providing more comprehensive and discriminative feature representations for emotion recognition.

#### 2.2.1. Feature Extraction

Studies have shown that the model achieves the best performance in emotion recognition when the time window length is set to 2 s [31]. Therefore, to capture signal characteristics at critical time points, this study employs non-overlapping 2 s time windows to segment both EEG and fNIRS signals [28]. The windowed signals undergo modality synchronization and baseline correction to obtain multimodal input samples. EEG and fNIRS signals both have a duration of 4694 s. After preprocessing and segmentation, 2347 time windows were obtained for each modality.

EEG signals reflect variations in brain activation levels and rhythmic patterns under different emotional stimuli, with distinct emotional states exhibiting different energy distributions across EEG frequency bands [32]. These distributional characteristics hold significant importance for emotion recognition. First, the EEG time signals are downsampled to 200 Hz, and a bandpass filter is applied for noise reduction. Power spectral density (PSD) and differential entropy (DE) features are then extracted from five commonly used frequency bands: *δ* (0.5–4 Hz), *θ* (4–8 Hz), *α* (8–13 Hz), *β* (13–30 Hz), and *γ* (30–50 Hz). PSD describes the energy distribution of the signal across different frequencies, while DE reveals nonlinear variations in EEG signals in the frequency domain. Given an EEG signal *x*, the calculations of PSD and DE are as follows.

(1) $$PSD={\left|\int_{-\infty }^{+\infty }x(\tau )e^{-j2\pi f\tau }d\tau \right|}^{2}$$

(2) $$DE=\frac{1}{2}\log(2\pi e\sigma^{2})$$

where *f* denotes frequency, *e* represents Euler’s number, and *σ*^2^ denotes the variance of *x*. Consequently, for each time window, PSD and DE features are obtained for each channel and frequency band, resulting in feature matrices of dimensions 2347 × 62 × 5 (samples × channels × input features). In the spatiotemporal dynamic modeling of EEG time-domain data, the temporal signals are downsampled to 11 Hz to align with the frequency of fNIRS signals, yielding time-domain features of size 2347 × 62 × 22 (samples × channels × input data). Among them, the DE and PSD features of EEG are fed into the FGCN module, while the temporal domain input of EEG is fed into the corresponding TCNN module.

fNIRS signals reflect local metabolic changes in the cerebral cortex and exhibit high spatial resolution, with a frequency range concentrated between 0.01 and 0.2 Hz. Due to the lack of clear frequency band delineation, this study primarily focuses on the spatiotemporal dynamic responses of fNIRS signals along the temporal dimension. The fNIRS temporal samples are constructed with a size of 2347 × 18 × 22 (sample channels input data) and fed into the corresponding TCNN module for feature extraction.

#### 2.2.2. FGCN Module

**Figure 3:**
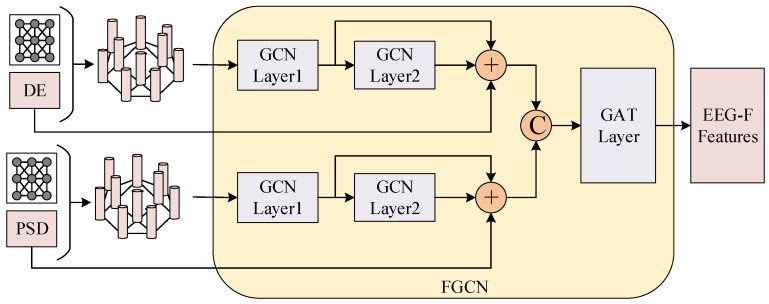
The framework of FGCN module.

The FGCN module integrates GCN and graph attention network (GAT) to focus, respectively, on the local topological structure and global information connectivity of electroencephalogram signals. GCN constructs an adjacency matrix based on the principle that neural connection strength attenuates with distance, capturing local topological features among nodes. GAT further incorporates an attention mechanism to adaptively adjust connection weights between channels, thereby modeling global cooperative relationships among channels. The framework of FGCN module is illustrated in Figure 3.

First, following the principle proposed by Salvador et al. [33] that neural connection strength decays inversely with the square of the physical distance, a physiologically meaningful adjacency matrix is constructed. The calculation formula for the adjacency matrix is as follows [22].

(3) $$A_{ij}=\left\{\begin{array}{l} 1,A_{ij}\geq 1\\ A_{ij}=\frac{\delta }{d_{ij}^{2}},0.1<A_{ij}<1\\ 0.1,A_{ij}\leq 0.1 \end{array}\right.$$

where *δ* denotes the scaling factor with its value determined as 9 for best results, *d_ij_* denotes the 3D distance between two channels. The initial adjacency matrix *A_ij_* is normalized and is treated as a learnable parameter throughout the model training process.

The extracted DE and PSD features are combined with the aforementioned adjacency matrix to construct two graph datasets for subsequent GCN learning. The update formula of the GCN network is as follows.

(4) $$\left\{\begin{array}{l} H^{\left(l+1\right)}=\sigma ({\tilde{D}}^{-\frac{1}{2}}\tilde{A}{\tilde{D}}^{\frac{1}{2}}H^{\left(l\right)}W^{l})\\ {\tilde{D}}_{ii}=\left(\sum_{j}{\tilde{A}}_{ij}\right)\\ \tilde{A}=A+I \end{array}\right.$$

where *H*^(*l*)^ denotes the node features at layer *l*, *H*^(0)^ represents the original input features, the rectified linear unit (ReLU) activation function is employed here, *W*^(*l*)^ denotes the learnable weight matrix at layer *l* for linear transformation. $\tilde{D}$ is the degree matrix of $\tilde{A}$, with ${\tilde{D}}_{ii}$ representing the diagonal elements of $\tilde{D}$, and *I* denotes the identity matrix. The identity matrix is added to the original adjacency matrix to enable each node to retain its own features.

The DE and PSD features are separately processed through a two-layer GCN to capture local topological features of the nodes. Let *X_DE_* and *X_PSD_* denote the DE and PSD features, respectively. After passing through the two-layer GCN, the DE features yield $H_{DE}^{\left(1\right)}$ and $H_{DE}^{\left(2\right)}$; similarly, the PSD features yield $H_{PSD}^{\left(1\right)}$ and $H_{PSD}^{\left(2\right)}$. To preserve the original frequency-domain features and fuse the local representations from different layers, we concatenate the input and the two-layer outputs for each branch along the feature dimension. The final fused feature *H* is then obtained as follows:

(5) $$\left\{\begin{array}{l} H_{DE}=concat(X_{DE},H_{DE}^{\left(1\right)},H_{DE}^{\left(2\right)})\\ H_{PSD}=concat(X_{PSD},H_{PSD}^{\left(1\right)},H_{PSD}^{\left(2\right)})\\ H=concat(H_{DE},H_{PSD}) \end{array}\right.$$

To integrate the complementary frequency-domain information from both features and model global cooperative relationships among channels, this study concatenates the outputs and feeds the fused features into the GAT. GAT dynamically assigns weights based on the features between nodes. The node feature update calculation formulas are as follows, where Equation (6) computes the attention scores, and Equation (7) represents the node feature update.

(6) eij=Leaky Re LU(aTWh→i||Wh→j)

(7) h→′=Leaky Re LU(∑soft max(eij)Wh→j)

where *h_i_* and *h_j_* represent the input features of nodes *i* and *j*, respectively. *W* is the linear transformation matrix, a is a learnable weight vector and ‖ denotes concatenation. Regularization and dropout layers are incorporated into the network to prevent overfitting, ultimately outputting EEG features that integrate both local and global information.

For the DE features, the first GCN layer maps the node feature dimension from 5 to 10, and the second GCN layer further maps it from 10 to 15. We employ skip-connection feature concatenation to fuse representations from different layers, yielding a 30-dimensional high-level frequency-domain representation for each node. The PSD features are processed in the same manner to obtain a corresponding 30-dimensional representation. Finally, the high-level DE and PSD representations are concatenated along the feature dimension to form a 60-dimensional input for each node, which is then fed into a six-head attention-based GAT network. This network preserves the local topological characteristics based on distance priors while simultaneously capturing inter-channel-interaction relationships among distant channels through the attention mechanism, thereby providing more physiologically interpretable frequency-domain feature representations for subsequent multimodal fusion.

#### 2.2.3. TCNN Module

**Figure 4:**
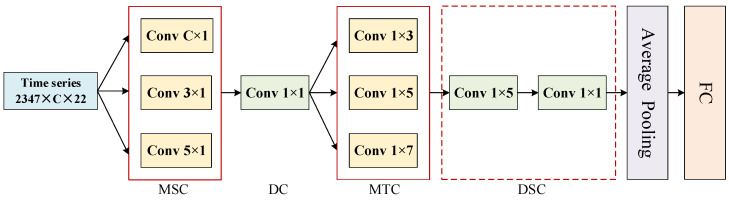
The framework of TCNN module.

Inspired by the lightweight EEG recognition network EEGNet proposed by Lawhern et al. [34] and the fNIRS-based emotion recognition network fNIRSNET introduced by Pandey et al. [29], this study designs a TCNN module tailored for EEG and fNIRS signals, as shown in Figure 4. The model primarily comprises multi-scale spatial convolutional layers (MSC), pointwise convolution (DC), multi-scale temporal convolutional layers (MTC), and depthwise separable convolution (DSC). Its core design principle lies in hierarchically modeling temporal variation trends with spatial context as a precondition. Detailed parameter specifications of the TCNN module are provided in Table 1.

The TCNN module first performs multi-scale spatial convolution to construct inter-channel cooperative responses. The multi-scale spatial convolutional layer consists of one global convolutional layer and two local convolutional layers. For different input signals, the kernel size of the global convolutional layer varies: it is set to 62 × 1 for EEG signals and 18 × 1 for fNIRS signals. These are combined with local convolutional layers to model spatial information from locally adjacent channels and global cooperative activations across channels, where local convolutional kernel sizes are selected as 3 × 1 and 5 × 1. Subsequently, a pointwise convolutional layer compresses high-dimensional spatial information to eliminate redundancy and emphasize dominant response channels. The compressed features are then fed into multi-scale temporal convolutional layers to hierarchically model variation trends along the temporal dimension. Finally, a depthwise separable convolutional layer extracts temporal features at different scales. All convolutional layers are followed by layer normalization and square activation functions to enhance nonlinearity and mitigate overfitting. The TCNN module achieves layer-by-layer extraction and fusion of spatiotemporal features, thereby improving the model’s expressive and generalization capabilities.

EEG time-domain signals and fNIRS time-domain signals are separately input into the TCNN module to extract time-varying response characteristics, yielding EEG spatiotemporal features and fNIRS spatiotemporal features.

#### 2.2.4. CAF Module

**Figure 5:**
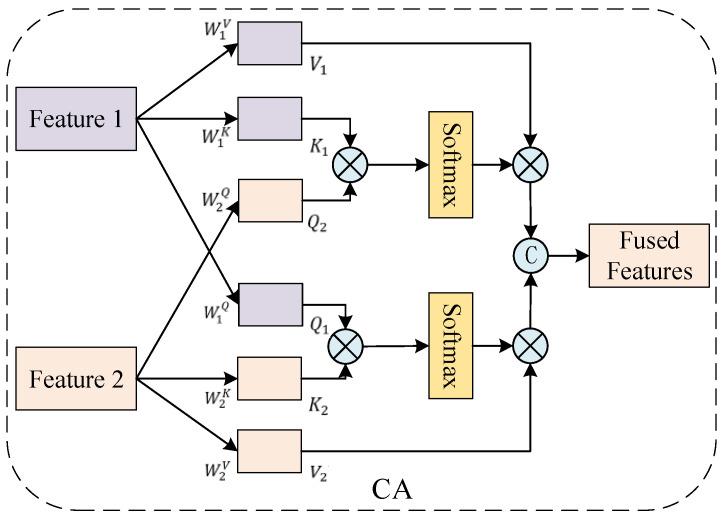
CA module.

To achieve deep interactive fusion between modalities, this study designs a feature fusion network based on a dual-path cross-attention mechanism, termed the CAF model. The dual-path cross-attention mechanism maps two distinct feature sets into a shared attention space, performs information selection through attention, and facilitates interaction and information complementarity between the two inputs. The dual-path cross-attention model (CA) is illustrated in Figure 5.

Let the features of the two input signals be denoted as *H*_1_ and *H*_2_, respectively. Linear transformations are applied to each to generate three sets of vectors:

(8) $$Q_{2}=H_{2}W_{2}^{Q},K_{1}=H_{1}W_{1}^{K},V_{1}=H_{1}W_{1}^{V}$$

where *Q*_2_ corresponds to the query vector of *H*_2_, and *K*_1_ and *V*_1_ are the key and value vectors of *H*_1_, respectively. The attention output for *H*_1_ is calculated as

(9) $$H_{1}^{\prime }=soft\;\max(\frac{Q_{2}K_{1}}{\sqrt{D}})V_{1}$$

Similarly, we perform a symmetric operation on the other feature to obtain the attention-processed feature $H_{2}^{\prime }$. The two outputs are then concatenated, yielding the fused feature:

(10) $$H=concat(H_{1}^{\prime },H_{2}^{\prime })$$

The CAF focuses on three distinct features: EEG frequency-domain features, EEG spatiotemporal features, and fNIRS spatiotemporal features. Attention-based interactions are performed pairwise among these three features, yielding six attention outputs. This approach achieves triple interactive fusion of frequency-domain channel-interaction features and time-domain spatiotemporal dynamic responses. The concatenated outputs are subsequently fed into the following classification module as an integrated input.

#### 2.2.5. Emotion Classification

To fully exploit the emotional discriminative information embedded in the multimodal fusion features, these features are fed into a multilayer perceptron (MLP) for classification. First, the input features are mapped through a linear layer, followed by a leaky rectified linear unit (LeakyReLU) activation function and a dropout layer to enhance the model’s generalization capability. Subsequently, a linear layer further reduces dimensionality and applies activation, ultimately performing classification. By stacking multiple linear transformations and nonlinear activations, discriminative classification of multimodal emotion fusion features is achieved.

## 3. Results

### 3.1. Experimental Settings and Evaluate

The hardware configuration employed in this experiment includes an Intel(R) Core(TM) i7-14650HX CPU and an NVIDIA GeForce RTX 4060 Laptop GPU. The operating system is Windows 11, and the software environment comprises Python 3.8 and CUDA 12.7. The experimental data are derived from TYUT, involving data from 50 participants. A 5-fold cross-validation method is adopted. Model performance was assessed by the average accuracy and standard deviation across all participants. The AdamW optimizer is utilized with a learning rate set to 0.001, a batch size of 128, and 100 training epochs. The cross-entropy loss function is selected, and five-fold cross-validation is applied to the data to ensure robust model performance and generalization capability.

**Figure 6:**
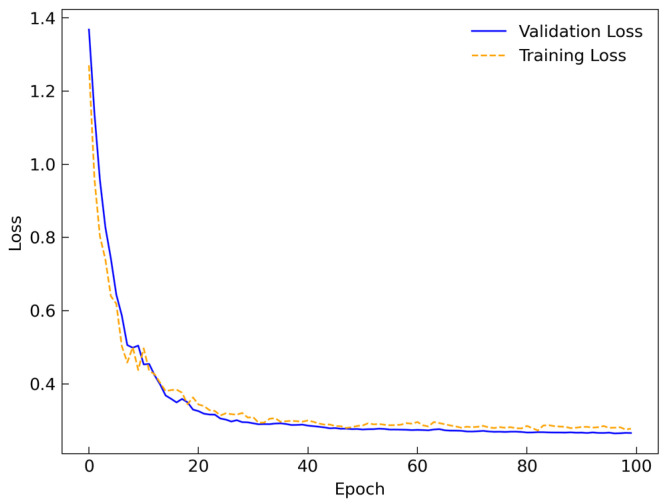
Training and validation loss curves.

Figure 6 presents the loss curves of the proposed module during training and validation. The loss curves indicate a rapid decline in loss during the early stages and stabilization in the later training phases, demonstrating the model’s strong generalization ability.

### 3.2. Performance Analysis of the FGCN-TCNN-CAF Module

To validate the performance of the proposed FGCN-TCNN-CAF module, experimental tests are conducted from three perspectives: single-view effectiveness, complementary advantages of dual modalities, and multi-view fusion efficacy. First, the classification performance of different EEG input views is compared, as shown in Table 2. The classification accuracy achieved using EEG frequency-domain features alone surpasses that of time-domain features, indicating that inter-channel-interaction modeling in the frequency domain better reveals emotional variations. When both features are jointly modeled, the recognition accuracy increases to 94.36%, demonstrating the effectiveness of multi-feature fusion within a single modality.

**Figure 7:**
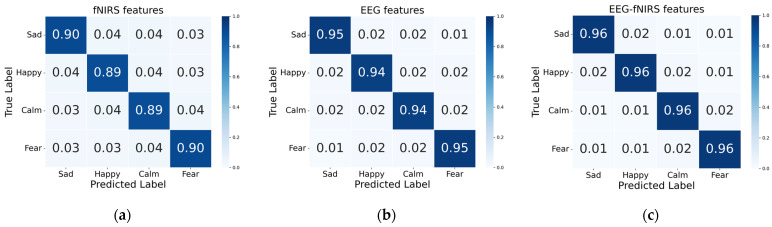
Confusion matrices for emotion recognition with different features: (**a**) fNIRS features; (**b**) EEG features; (**c**) EEG-fNIRS features.

Furthermore, we compare the fusion results of EEG frequency domain and time domain features with the complementary fusion outputs of fNIRS time-domain and EEG-fNIRS combinations. Table 3 presents the classification performance across different modal data. The results in Table 3 reveal that the recognition accuracy of the fNIRS unimodal model is lower than that of the EEG model. However, the emotion recognition accuracy improves when EEG and fNIRS modalities are jointly utilized, showing an increase of 1.73% compared to the EEG unimodal model and 6.65% compared to the fNIRS unimodal model. Additionally, the standard deviation of bimodal emotion recognition is lower than that of unimodal models, indicating that multimodal emotion recognition exhibits enhanced stability and robustness. Figure 7 displays the confusion matrices for emotion using different features. As shown in Figure 7, the emotion recognition accuracy for each category in the bimodal model shows improvement compared to unimodal models, with reduced misclassification across all four emotion types. The physiological complementarity between EEG and fNIRS provides more comprehensive information support for emotion recognition, thereby enhancing model stability and discriminative capability.

To evaluate the further performance improvement facilitated by multi-view information fusion, we investigate combination strategies involving three views of EEG and fNIRS, with results presented in Table 4. The average recognition accuracy achieved with the three-view fusion of EEG and fNIRS is 0.74% higher than that of the joint view of EEG frequency-domain and fNIRS time-domain features, and 2.07% higher than that of the joint view of EEG time-domain and fNIRS time-domain features. These findings demonstrate that the proposed multi-view emotion recognition approach fully leverages the complementary information between EEG and fNIRS, further validating the enhanced expressive power of multimodal multi-view integration for emotion feature representation.

### 3.3. Validation of the Spatial-First-Temporal-Later Paradigm in TCNN Module

To validate the impact of the “spatial-first, temporal-second (SFTS)” convolutional modeling strategy in the proposed TCNN network on whole model performance, we conduct comparative experiments on convolutional order. Specifically, while keeping the rest of the network structure unchanged, we reversed the convolutional order to “temporal-first, spatial-second (TFSS)”, which means performing multi-scale temporal convolution before spatial convolution. We conduct classification tests under different input views and evaluate the modeling order using multiple metrics. In addition to accuracy, we further introduce precision, recall, and F1-score as complementary metrics. All metrics are calculated using the macro-average method. The experimental results are presented in Table 5. When using only EEG time-domain signals, the module achieves an accuracy of 91.55%, representing an improvement of 18.93% over the TFSS structure, while precision, recall, and F1-score reach 91.56%, 91.61%, and 91.58%, respectively. When using only fNIRS time-domain signals, the SFTS strategy improves accuracy by 3%, with consistent improvements across all comprehensive metrics. Under the joint view of both EEG and fNIRS time-domains, accuracy increases by 7.05%, with precision, recall, and F1-score all stabilizing around 94%. The overall model accuracy reaches 96.09%, an improvement of 1.65% over TFSS’s 94.44%, with all metrics exceeding 96%. These results demonstrate the effectiveness of the spatial-first, temporal-second model framework. The initial multi-scale spatial convolution models inter-channel cooperative relationships, exhibiting stronger feature compression capability. Subsequently, multi-scale temporal convolution captures the dynamic evolution of spatial cooperative relationships over time, thereby laying a foundation for subsequent fusion and classification.

### 3.4. Ablation Study

To validate the effectiveness of the proposed FGCN-TCNN-CAF module, comparative experiments are conducted on each module of the model, with recognition results presented in Table 6. The baseline model GCN refers to a conventional two-layer graph convolutional neural network, achieving a recognition accuracy of 90.16%. FGCN incorporates residual connections to mitigate the gradient vanishing issue in deep networks. By integrating a GAT network that models the importance of inter-channel relationships, the module substantially strengthens the representation of frequency-domain channel-interaction features. This approach yields a 2.62% improvement in recognition accuracy. The FGCN-TCNN module further extends the FGCN module by incorporating the TCNN module, leading to an accuracy improvement of 2.78%. The TCNN module effectively captures spatiotemporal features of time-series signals, complementing the frequency-domain features extracted by the FGCN module to construct multi-level information spanning spatiotemporal, frequency, and channel dimensions, thereby providing more comprehensive feature representations for subsequent classification tasks. The complete FGCN-TCNN-CAF module augments this architecture with a cross-attention fusion network, achieves triple interactive fusion of EEG-fNIRS spatiotemporal, frequency, and inter-channel correlations, and attains the highest recognition accuracy of 96.09%.

Furthermore, the FGCN module achieved a significant improvement in accuracy at the cost of only a modest increase in parameters and computational time, demonstrating the effectiveness of residual connections and graph attention mechanisms. The TCNN module further enhanced the model by incorporating spatiotemporal modeling capabilities, while the CAF module facilitated interactive fusion across modalities. These components contributed to the progressive optimization of model performance. Compared to the baseline GCN, the complete FGCN-TCNN-CAF model achieved a 5.93% increase in accuracy and improved prediction stability, despite a 49.6% growth in the number of parameters and a 3.5-fold increase in the average processing time per subject. Although computational costs rose, the per-subject processing time remained manageable at approximately two minutes. This represents a favorable balance between efficiency and accuracy for emotion recognition tasks, demonstrating that our model maximizes performance gains with acceptable complexity.

**Figure 8:**
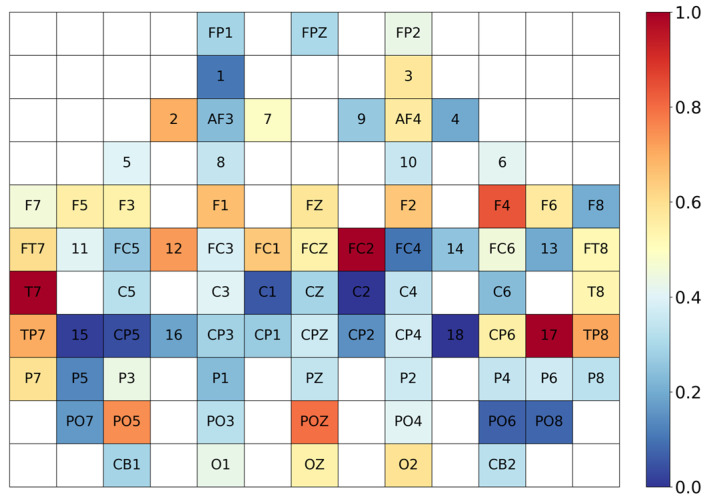
The visualized weight distribution of the CAF module. In the diagram, 1–18 represent fNIRS channels, while the remaining channels denote EEG electrodes.

To present the interpretability of the CAF module, we spatially mapped its six output pathways. Figure 8 illustrates the attention weight distribution of the CAF module, visually presenting the varying importance of different channels in emotion recognition. EEG has a higher number of electrode channels compared to fNIRS, and their positions do not overlap, enabling effective complementarity. Moreover, channels in the frontal and temporal lobes are significantly activated, which aligns with the physiological mechanisms underlying emotion processing. The CAF module achieves triple interaction and fusion across temporal, frequency and channel dimensions, contributing to enhanced recognition performance.

### 3.5. Comparative Evaluation of FGCN-TCNN-CAF Module

To validate the effectiveness of the FGCN-TCNN-CAF module, the proposed model is compared with other emotion recognition modules. All models are fed with identical EEG and fNIRS signals and evaluated under the same experimental conditions for a fair comparison. The recognition results of each model presented in Table 7. CTNet [16] comprises a CNN module for processing time series and an attention-based Transformer module. 2DCNN [17] maps node features and extracts spatiotemporal features through 2D convolution. fNIRSNET [29] utilizes convolutional neural networks to generate and fuse multi-view spatiotemporal features. ST-CapsNet [18] integrates spatial and temporal attention with capsule networks to learn more discriminative features. TC-ResNet [27] combines a temporal convolutional network with a residual network for temporal feature extraction. As shown in Table 7, the proposed FGCN-TCNN-CAF module achieves the highest recognition accuracy among the listed methods while exhibiting a smaller standard deviation. By modeling frequency-domain channel-interaction features and employing a heterogeneous parallel network for cross-modal time-domain spatiotemporal features, combined with a cross-attention fusion network, our approach effectively integrates the complementary information of EEG and fNIRS, significantly enhancing emotion recognition performance.

### 3.6. Visualization

**Figure 9:**
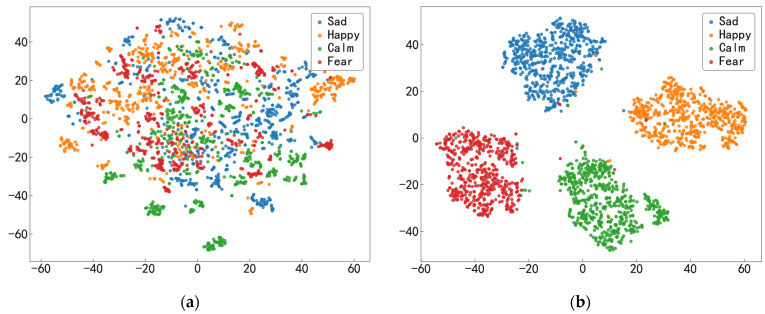
t-SNE visualization of feature distributions. (**a**) Input features; (**b**) fused features after model learning. Different colors denote different emotion categories.

To more intuitively illustrate the impact of the proposed model on feature representations, we employ t-SNE to project the model’s input and output features into a two-dimensional space for visualization, as shown in Figure 9. The input features consist of DE, PSD, and time-series features from EEG signals, along with time-series features from fNIRS signals, while the output features are the fused representations generated by the FGCN-TCNN-CAF model. Figure 9a presents the distribution of the input features, and Figure 9b shows the distribution of the fused features after model learning.

As observed in Figure 9a, samples from different emotion classes are highly mixed with substantial overlap, indicating that the input features are disorganized and difficult to distinguish. In contrast, after being processed by the model, samples from different emotion categories form more compact intra-class clusters and exhibit a clearer inter-class separation trend, with only a few outliers near the cluster boundaries. This distribution suggests that the model successfully extracts more discriminative emotional representations. These visualization results are consistent with the improvement in classification performance, further indicating the effectiveness of the FGCN-TCNN-CAF module for EEG–fNIRS multimodal emotion recognition tasks.

### 3.7. Sensitivity Analysis

**Figure 10:**
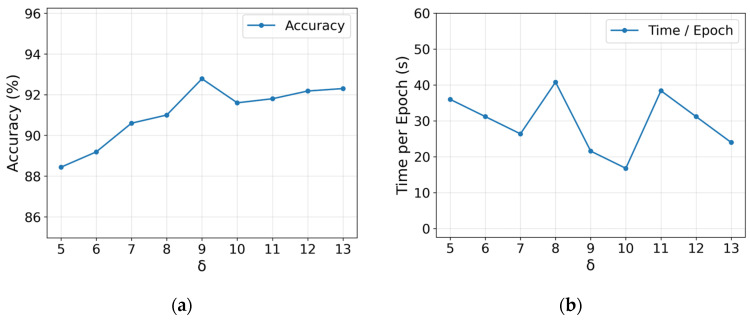
Impact of the scaling factor *δ* on FGCN performance. (**a**) Effect on classification accuracy; (**b**) effect on training time per epoch.

To evaluate the impact of the scaling factor *δ* (see Equation (3)) in the construction of the adjacency matrix on the FGCN module, we conducted experiments with different *δ* values while keeping other parameters unchanged. The results are shown in Figure 10. Figure 10a,b illustrate the classification accuracy and the average runtime per epoch of FGCN under different *δ* values, respectively.

As shown in Figure 10a, as *δ* increases, the model’s classification accuracy first improves and then stabilizes, reaching the highest accuracy of 92.78% for *δ* equal to 9. According to Equation (3), when *δ* is small, the edge weights are generally too small, resulting in insufficient connection strength in the adjacency matrix, which limits information propagation. As *δ* continues to increase, some edge weights tend to saturate due to the truncation mechanism, weakening the weight differences caused by varying distances and causing the model’s accuracy to stabilize. As shown in Figure 10b, although the average runtime per epoch fluctuates under different *δ* values, *δ* equal to 9 achieves the optimal accuracy while maintaining relatively low training overhead. Therefore, considering both accuracy and efficiency, this paper adopts *δ* equal to 9.

## 4. Discussion

EEG offers high temporal resolution and can dynamically capture rapid changes in neural electrical activity, whereas fNIRS provides relatively high spatial resolution and reflects localized metabolic variations in the cerebral cortex. Their combination provides a natural spatiotemporal complementarity for emotion recognition. However, existing EEG-fNIRS multimodal approaches often struggle to jointly model the coupling among frequency-domain information, inter-channel relationships, and spatiotemporal features. Cross-modal fusion is also frequently limited to simple feature aggregation, which makes it difficult to learn fine-grained correspondences and complementary mechanisms between EEG and fNIRS. To address these issues, we propose FGCN-TCNN-CAF, which adopts a modality-specific modeling strategy. FGCN captures the non-Euclidean channel topology in the frequency domain, TCNN extracts spatiotemporal dynamic response features in the time domain, and CAF enables interactive cross-modal fusion so that the model can adaptively select and integrate complementary information from both modalities. Ablation experiments confirm the effectiveness of each module: when certain components are removed, both the model’s performance and stability decline, indicating that modality-specific modeling and cross-modal interaction are key factors for improving EEG–fNIRS fusion. Moreover, comparisons with other baseline models further suggest that the proposed strategy yields more effective results in terms of both accuracy and stability.

Despite these promising results, several limitations remain. First, due to the mismatch in sampling rates between EEG and fNIRS, we downsample the EEG time-series from 200 Hz to 11 Hz to achieve synchronization, which may lead to the loss of critical temporal information and thus potentially affect emotion recognition performance. Second, although we align the modalities using a 2 s synchronized time window, the physiological delay of fNIRS relative to EEG is not explicitly modeled, which may limit the fine-grained modeling of cross-modal coupling. Therefore, future work will explore more effective strategies for cross-modal sampling-rate alignment and information preservation, and introduce explicit cross-modal alignment mechanisms to further improve the model’s generalization ability and fusion effectiveness.

## 5. Conclusions

In this paper, we model the frequency-domain, spatial, and temporal features of EEG-fNIRS signals and propose a novel framework named FGCN-TCNN-CAF. Specifically, the FGCN module builds an initial adjacency matrix according to inter-channel physical distance and leverages a GAT to strengthen channel interactions and the TCNN module employs spatial convolution followed by temporal convolution to capture spatiotemporal patterns. Furthermore, the CAF module enables cross-modal interactive fusion to integrate complementary information from EEG and fNIRS. Ablation experiments demonstrate the contribution of each module. On the ENTER dataset, the proposed model achieves an accuracy of 96.09%, suggesting that the proposed differentiated modeling strategy effectively exploits EEG-fNIRS complementarity and provides a viable solution for EEG-fNIRS emotion recognition.

## Figures and Tables

**Table 1:** Detailed parameter of the TCNN module.

Layer Type		Size	Output	Layer Type	Size	Output
input	EEG	B × 62 × 22	B × 1 × 62 × 22	DC	1 × 1@60	B × 60 × 1 × 22
	fNIRS	B × 68 × 22	B × 1 × 18 × 22	MTC	1 × 3@7	B × 21 × 1 × 22
MSC	EEG	62 × 1@20	B × 60 × 1 × 22		1 × 5@7	
		3 × 1@20			1 × 7@7	
		5 × 1@20		DSC	1 × 5@21	B × 21 × 1 × 22
	fNIRS	18 × 1@20	B × 60 × 1 × 22		1 × 1@10	B × 10 × 1 × 22
		3 × 1@20		average pooling	1 × 5	B × 10 × 1 × 9
		5 × 1@20		FC	-	B × 90

**Table 2:** Impact of EEG feature modeling approaches on emotion recognition.

Input View	Acc (%)				
	Avg Acc (std)	Sad	Happy	Calm	Fear
EEG-T ^1^	91.55 (2.19)	92.64	90.64	90.94	92.02
EEG-F ^2^	92.78 (2.99)	92.99	92.73	92.92	92.46
EEG-F + EEG-T	94.36 (1.72)	94.70	93.89	94.19	94.68

^1^ EEG time domain, ^2^ EEG frequency domain.

**Table 3:** Comparative performance of different modalities for emotion recognition.

Modality	Acc (%)				
	Avg Acc (std)	Sad	Happy	Calm	Fear
EEG	89.44 (3.44)	89.79	89.29	88.69	90.08
fNIRS	94.36 (1.72)	94.70	93.89	94.19	94.68
EEG + fNIRS	96.09 (1.17)	96.42	95.71	95.94	96.30

**Table 4:** Impact of multi-view combination strategies for EEG and fNIRS on emotion recognition.

Input View	Acc (%)				
	Avg Acc (std)	Sad	Happy	Calm	Fear
EEG-T ^1^ + fNIRS-T ^3^	94.02 (1.78)	94.88	93.65	93.46	94.12
EEG-F ^2^ + fNIRS-T	95.35 (1.62)	96.21	95.07	95.04	95.07
EEG-F + EEG-T + fNIRS-T	96.09 (1.17)	96.42	95.71	95.94	96.30

^1^ EEG time domain, ^2^ EEG frequency domain, ^3^ fNIRS time domain.

**Table 5:** Impact of different spatiotemporal convolution orders on model performance.

Input View	Orders		Avg Acc (%)	Precision (%)	Reall (%)	F1-Score (%)
	SFTS ^4^ (Ours)	TFSS ^5^				
fNIRS-T ^1^		1	86.44 (3.76)	86.47	86.45	86.46
	1		89.44 (3.44)	89.47	89.45	89.46
EEG-T ^2^		1	72.62 (5.88)	72.76	72.71	72.71
	1		91.55 (2.19)	91.56	91.61	91.58
EEG-T + fNIRS-T		1	86.97 (3.62)	86.96	87	86.98
	1		94.02 (1.78)	94.03	94.05	94.04
EEG-F ^3^ + EEG-T + fNIRS-T		1	94.44 (2.05)	94.44	94.45	94.44
	1		96.09 (1.17)	96.09	96.1	96.09

^1^ fNIRS time domain, ^2^ EEG time domain, ^3^ EEG frequency domain, ^4^ “spatial-first, temporal-second” in TCNN module, ^5^ “temporal-first, spatial-second” in TCNN module.

**Table 6:** Comparison of ablation results.

Model	Acc (%)					Number of Parameters	Times (s)
	Avg Acc (std)	Sad	Happy	Calm	Fear		
GCN	90.16 (5.10)	90.15	90.71	88.45	91.33	1,127,844	35
FGCN	92.78 (2.99)	92.99	92.73	92.92	92.46	1,471,178	40
FGCN-TCNN	95.56 (1.30)	95.84	95.47	95.14	95.82	1,517,168	87
FGCN-TCNN-CAF	96.09 (1.17)	96.42	95.71	95.94	96.30	1,688,816	121

**Table 7:** Comparison with other emotion recognition models.

Model	Avg Acc (std)
CTNet [16]	86.84 (9.25)
2DCNN [17]	90.56 (4.96)
fNIRSNET [29]	92.95 (2.08)
ST-CapsNet [18]	94.01 (2.95)
TC-ResNet [27]	95.04 (1.91)
FGCN-TCNN-CAF(ours)	96.09 (1.17)

## Data Availability

The data presented in this study are available on request from the corresponding author due to privacy and ethical reasons. If you are interested in the dataset and want to use it, please download and fill out the license agreement (https://gitee.com/tycgj/enter, accessed on 12 June 2025) and send it to chenguijun@tyut.edu.cn. We will send you a download link through email after a review of your application.
